# HoloLens-Based Vascular Localization System: Precision Evaluation Study With a Three-Dimensional Printed Model

**DOI:** 10.2196/16852

**Published:** 2020-04-17

**Authors:** Taoran Jiang, Dewang Yu, Yuqi Wang, Tao Zan, Shuyi Wang, Qingfeng Li

**Affiliations:** 1 Department of Plastic and Reconstructive Surgery Shanghai 9th People’s Hospital, Shanghai Jiao Tong University School of Medicine Shanghai China; 2 School of Medical Instrument and Food Engineering University of Shanghai for Science and Technology Shanghai China

**Keywords:** augmented reality, HoloLens, perforator flap, vascular localization, reconstructive surgery, 3D Printing

## Abstract

**Background:**

Vascular localization is crucial for perforator flap transfer. Augmented reality offers a novel method to seamlessly combine real information with virtual objects created by computed tomographic angiography to help the surgeon “see through” the skin and precisely localize the perforator. The head-mounted display augmented reality system HoloLens (Microsoft) could facilitate augmented reality–based perforator localization for a more convenient and safe procedure.

**Objective:**

The aim of this study was to evaluate the precision of the HoloLens-based vascular localization system, as the most important performance indicator of a new localization system.

**Methods:**

The precision of the HoloLens-based vascular localization system was tested in a simulated operating room under different conditions with a three-dimensional (3D) printed model. The coordinates of five pairs of points on the vascular map that could be easily identified on the 3D printed model and virtual model were detected by a probe, and the distance between the corresponding points was calculated as the navigation error.

**Results:**

The mean errors were determined under different conditions, with a minimum error of 1.35 mm (SD 0.43) and maximum error of 3.18 mm (SD 1.32), which were within the clinically acceptable range. There were no significant differences in the errors obtained under different visual angles, different light intensities, or different states (static or motion). However, the error was larger when tested with light compared with that tested without light.

**Conclusions:**

This precision evaluation demonstrated that the HoloLens system can precisely localize the perforator and potentially help the surgeon accomplish the operation. The authors recommend using HoloLens-based surgical navigation without light.

## Introduction

High variability in perforator size and course, along with distorted anatomical landmarks after injury are the two key challenges in precisely predicting the location of the perforator in a vascular flap transfer procedure. Precisely locating the perforator can be difficult for new microsurgeons even without these key challenges. Computed tomographic angiography (CTA) has been used as a noninvasive and effective tool for vascular mapping to plan the positioning of perforator flaps [1,2] by revealing the characteristics of perforators, such as the caliber, length, and course [3]. Using the three-dimensional (3D) objects reconstructed by CTA data, the relation between the perforator and the underlying skeleton and nearby muscle and skin can be immediately identified. This enables the surgeon to identify the specific perforator anatomy of the patient. Accordingly, CTA is increasingly applied as a standard preoperative procedure in planning flap design, and its superiority over Doppler ultrasound has been well-established [4].

However, the information and 3D objects supplied by CTA are typically evaluated on a two-dimensional screen, which limits the ability to encompass the true depth and the possibilities for transferring detailed information onto the patient’s body during the operation. Augmented reality (AR) has emerged as a novel method to overcome these barriers. In AR, virtual objects are seamlessly combined with real information to generate a “see-through” image. In particular, AR allows for 3D objects, including the vasculature, bone, muscle, and skin, to be overlaid onto the patient’s body during the operation, serving as a guide to precisely and rapidly localize the perforator.

Without a head-mounted display (HMD), AR images can only be displayed on a monitor, which requires the surgeon to repeatedly switch his/her view between the monitor and operating field. This action can reduce the surgical precision for some delicate operations such as perforator localization. HoloLens, developed by Microsoft Corporation, is considered a very suitable AR HMD for surgical practice [5]. When wearing HoloLens, surgeons are able to see the AR images in a single view. Another key advantage of HoloLens is that it is not only a display system but is also a self-contained computer equipped with a camera and sensors, thereby avoiding the need to install any other hardware to realize the AR effect, which could further help to reduce the risk of contamination in the operating room.

HoloLens has been tentatively used in several surgical fields to date, including heart surgery [6], visceral surgery [7], and orthopedic surgery [8]. Most of these studies focused on the feasibility and performance of HoloLens; however, few studies have examined the precision of HoloLens in surgical practice. We previously verified the feasibility of AR-based perforator localization [9]. To improve the accuracy of this localization, HoloLens was applied to reduce the error caused by switching the view. In this study, the precision of this HoloLens-based perforator localization system was evaluated in a simulated operating room using a 3D printed model under different conditions, including with or without light, different intensities of light, and different visual angles.

## Methods

### Model Design

This study was conducted using a 3D printed model of a volunteer’s deep femoral artery and its branches. The volunteer underwent a thin-cut (0.1 mm) CTA scan (Siemens, Germany) with administration of a nonionic contrast agent (Omnipaque, China) and the DICOM files were imported into Mimics 19.0 software (Materialise, Belgium) to create a virtual model, including a vascular map and surrounding soft tissues. A holder was designed by CAD software 3D Max (Autodesk, Mill Valley, CA, USA) to fix the model so that it could be placed stably on the operating table. The holder could also be used to place a marker, which is very important during registration. The 3D virtual model with the vascular map, surrounding soft tissues, marker, and holder was then printed for use in precision analysis. Data of the virtual model of the vascular map and marker were then input into HoloLens.

### Augmented Reality Effect Workflow

The localization app was written within the Unity framework (Unity Technologies, San Francisco, CA, USA), which is a custom-developed HoloLens C# Universal Windows Platform app. The initial registration of the virtual model for real-world application was realized using a marker. Once launched, the app started the HoloLens built-in camera to acquire the view of the operative field. When the app finds the marker in the view, it registers the real marker with the virtual marker. The relative position between the virtual marker and the virtual model was the same as that between the real marker and the 3D printed model; consequently, the 3D printed model was registered with the virtual model at the time of registration of the real marker and virtual marker. After registration, the app generated AR images that were projected into the optical see-through lens incorporated in HoloLens. By tracking the position of the real marker though the camera, the position and angle of the virtual model automatically change according to the wearer’s perspective to obtain registered AR images in real time. The complete workflow was as follows. First, the surgeon wore the HoloLens and launched the app. Second, the app registered the virtual model and the real world, and generated the appropriate AR images. Finally, the wearer localized the vasculature under guidance of the AR images.

### Localization Precision Analysis

**Figure 1:**
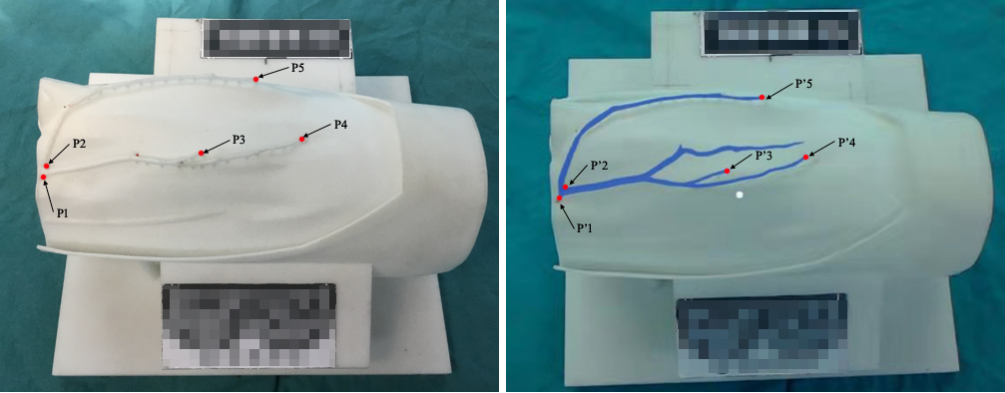
Three-dimensional printed model of the vascular map (A) and the augmented reality image with the virtual model of the vasculature overlapped onto the printed model (B). P1-P5, 5 points on the three-dimensional printed vascular map selected for precision analysis; P1’-P5’, 5 corresponding points on the virtual model.

**Figure 2:**
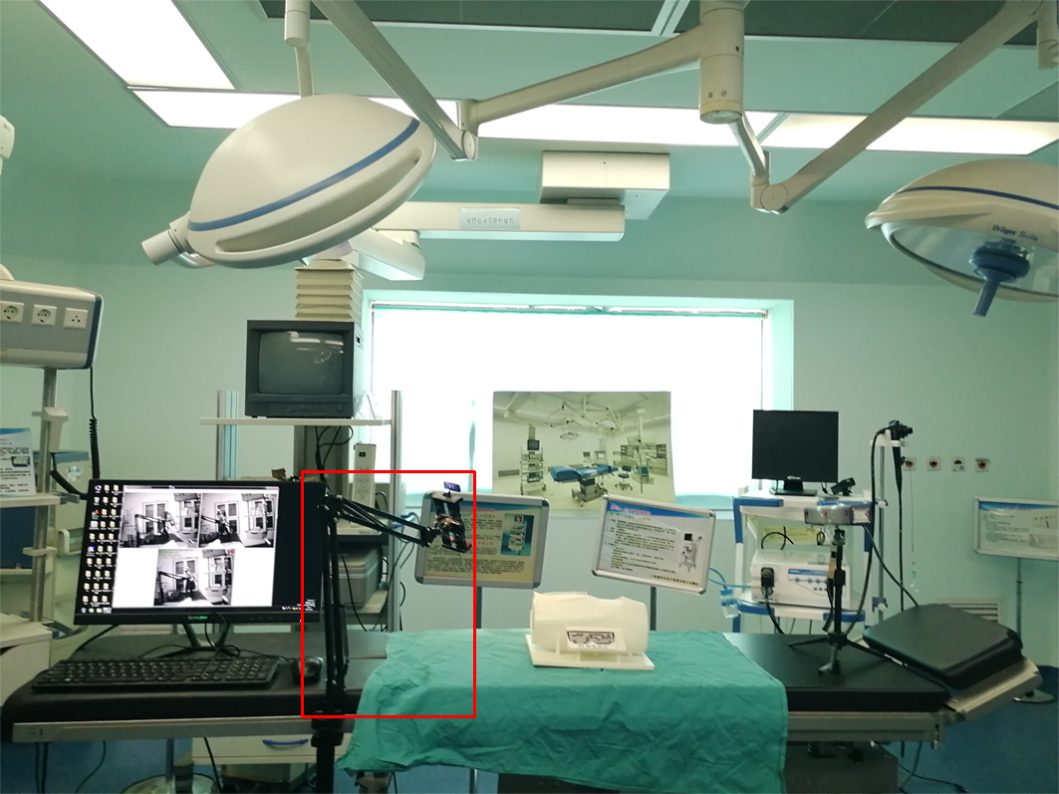
The simulated operating room used for the test. The probe was held by a mechanical arm.

Five pairs of points on the vascular map that could be easily identified on the 3D printed model and virtual model were selected for precision analysis (Figure 1). The coordinates of these pairs of points were recorded by a Micron Tracker tracking device (Claron Technology, Toronto, ON, Canada), which can be used to detect and calculate the space location through a probe (distance error<0.2 mm). To eliminate the error caused by hand tremor, a mechanical arm was used to hold the probe (Figure 2).

**Figure 3:**
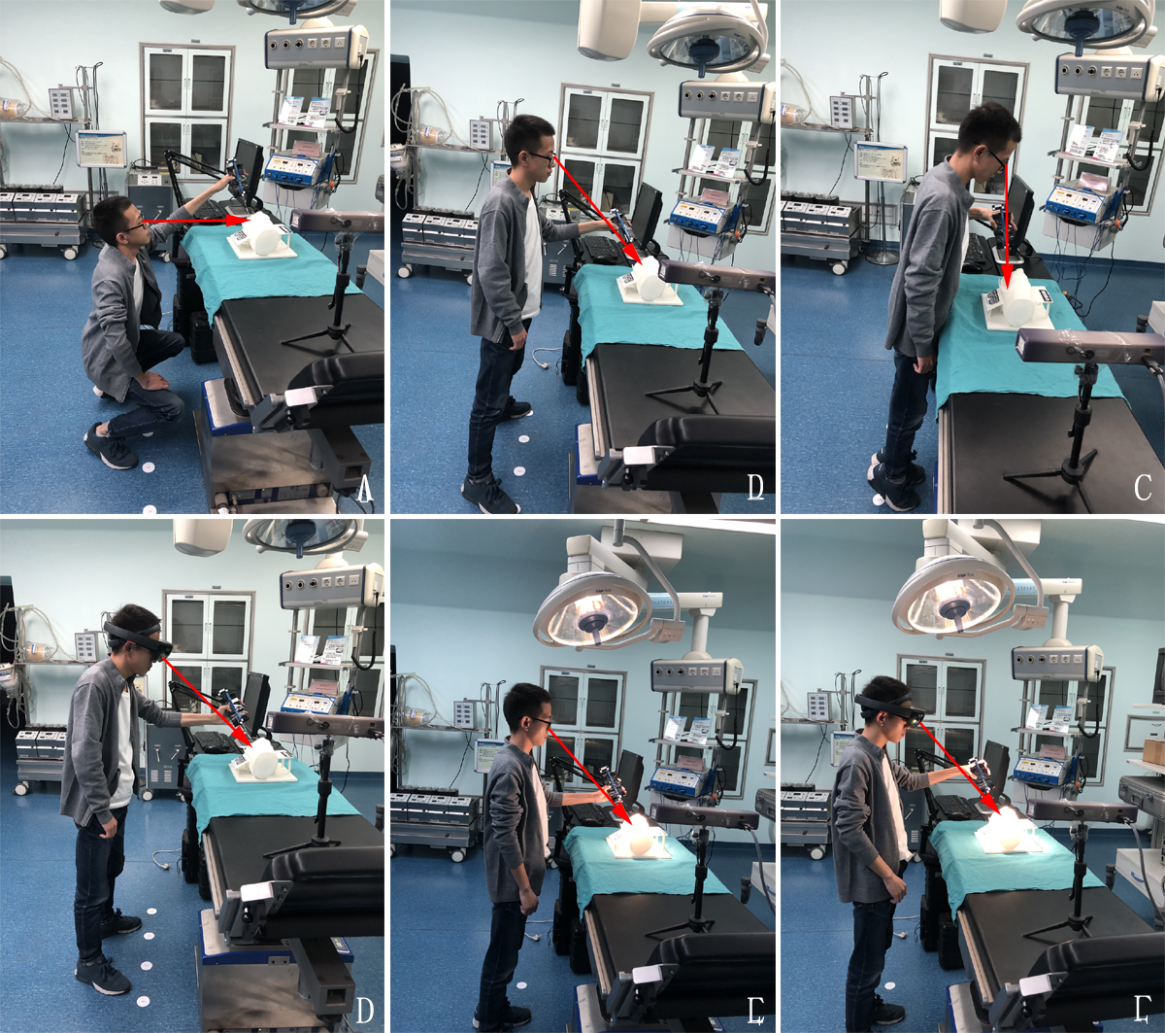
The coordinates of points were recorded by a tracking device (Micron Tracker) under different conditions. (A) The coordinates of points on the three-dimensional (3D) printed model were detected under the horizontal visual angle without light. (B) The coordinates of points on the 3D printed model were detected under the oblique visual angle without light. (C) The coordinates of points on the 3D printed model were detected under the vertical visual angle without light. (D) The coordinates of points on the virtual model were detected under the oblique visual angle without light. (E) The coordinates of points on the 3D printed model were detected under the oblique visual angle with light. (F) The coordinates of points on the virtual model were detected under the oblique visual angle with light.

The tests were carried out by 7 operators in a simulated operating room under different conditions, including with or without light, different intensities of light, and different visual angles (Figure 3). First, the coordinates of tested points in the 3D printed model were recorded without superimposing the virtual model. Then, the AR image was generated, and the coordinates of tested points in the virtual model were recorded. The distance between the corresponding points was then calculated with the following formula, and the distances were averaged to determine the overall error:

Distance = square root ([X1 – X2]^2^ + [Y1 – Y2]^2^ + [Z1 – Z2]^2^)

Where X1, Y1, and Z1 are the coordinates of the point in the 3D printed model, and X2, Y2, and Z2 are the coordinates of the corresponding point in the virtual model.

**Figure 4:**
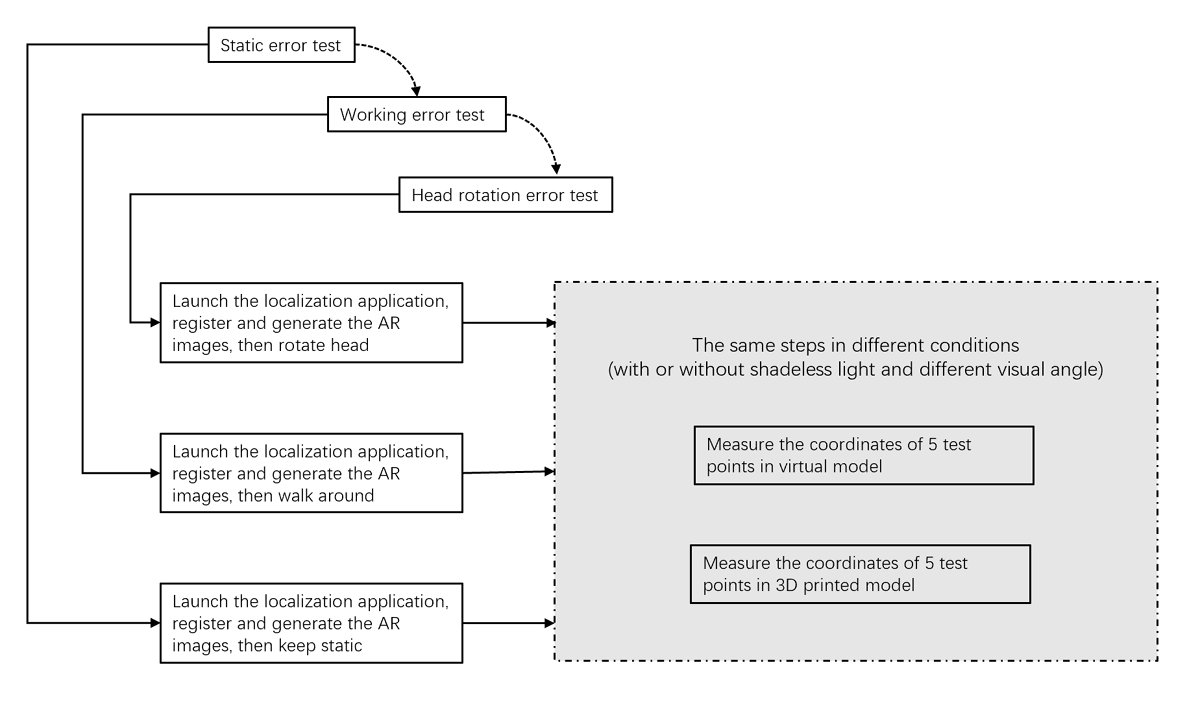
Workflow of the test. AR: augmented reality; 3D: three-dimensional.

Two types of errors were considered in this study: static error and motion error. The latter included the error caused by head rotation (ie, error caused by rotating the head to look around and then returning the head to the initial state without body motion) and error caused by walking (ie, walking around the operation room and then returning to the initial position). Figure 4 shows the workflow of the test. The detailed content of the test is shown in Table 1.

**Table 1:** Detailed content of the performance test.

Error value	Concrete content
SE-V	Average static error of 5 test points without light under vertical observation
SE-O	Average static error of 5 test points without light under oblique observation (45°)
SE-H	Average static error of 5 test points without light under horizontal observation
SE-(V/O/H)-L	Average static error of 5 test points under low light intensity and the best observation angle
SE-(V/O/H)-M	Average static error of 5 test points under medium light intensity and the best observation angle
SE-(V/O/H)-H	Average static error of 5 test points under high light intensity and the best observation angle
SE-(V/O/H)-SH	Average static error of 5 test points under superhigh light intensity and the best observation angle
HE-(V/O/H)	Average head rotation error of 5 test points without light under the best observation angle
WE-(V/O/H)	Average walking error of 5 test points without light under the best observation angle

### Statistical Analysis

Continuous variables are presented as the mean and SD and were analyzed by paired Student *t* tests. A *P* value less than .05 was considered to represent a statistically significant difference.

## Results

The results are summarized in Tables 2-5. The static error was first recorded under different visual angles without light, demonstrating no significant differences (horizontal vs oblique *P*=.25; horizontal vs vertical *P*=.25; oblique vs vertical *P*=.10; Table 2).

Since the oblique visual angle was similar to the surgeon’s visual angle, further tests were performed under the oblique visual angle, and the static errors were recorded with different light intensities. There was no significant difference among the errors tested under these light intensities (low vs medium *P*=.25; low vs high *P*=.32; low vs superhigh *P*=.08; medium vs high *P*=.32; medium vs superhigh *P*=.43; high vs superhigh *P*=.09; Table 3).

However, the difference between the errors tested with and without light was statistically significant (*P*=.02; Table 4).

Finally, the motion errors under an oblique angle without light were recorded. Compared with the static error, there was no significant difference in either the head rotation (*P*=.25) or walking (*P*=.30) motion errors (Table 5).

**Table 2:** Static error tested without light under different visual angles (mm).

Operator	Visual angle, degrees		
	0 (Horizontal)	45 (Oblique)	90 (Vertical)
1	1.28	0.63	1.63
2	1.80	1.47	1.28
3	0.74	1.01	1.36
4	1.83	1.3	1.73
5	1.94	1.63	1.43
6	1.26	1.45	2.31
7	1.44	1.96	1.67
Mean (SD)	1.47 (0.42)	1.35 (0.43)	1.63 (0.34)

**Table 3:** Static error under different light intensities (mm).

Operator	Light Intensity			
	Low	Medium	High	Superhigh
1	2.70	1.33	1.91	1.93
2	2.82	4.00	2.55	3.25
3	3.93	4.23	5.03	4.60
4	1.74	1.99	1.74	2.27
5	2.97	2.26	3.05	2.99
6	4.69	6.39	4.89	5.31
7	1.11	1.77	1.54	1.94
Mean (SD)	2.85 (1.21)	3.14 (1.81)	2.96 (1.46)	3.18 (1.32)

**Table 4:** Static error with or without light (mm).

Operator	With light (low intensity)	Without light
1	2.70	0.63
2	2.82	1.47
3	3.93	1.01
4	1.74	1.3
5	2.97	1.63
6	4.69	1.45
7	1.11	1.96
Mean (SD)	2.85 (1.21)	1.35 (0.43)

**Table 5:** Motion errors under an oblique angle without light (mm).

Operator	Walking	Head rotation	Static
1	1.06	1.34	0.63
2	2.28	0.84	1.47
3	1.59	3.35	1.01
4	1.02	1.50	1.3
5	1.35	1.44	1.63
6	1.38	1.28	1.45
7	1.5	1.74	1.96
Mean (SD)	1.45 (0.42)	1.50 (0.80)	1.35 (0.43)

## Discussion

### Principal Findings

We previously verified the feasibility of AR-based perforator localization. Under the guidance of localization, a surgeon successfully dissected a beagle’s thoracodorsal artery perforator without any prior anatomical knowledge of the dog [9]. The mean error of the localization system was 3.5 mm. However, the HMD (nVisor ST60, NVIS Company, USA) used in this previous system was merely an AR display without any other function, and thus this system had to be equipped with a computer workstation and a 3D camera, which increases the complexity of the system as well as the risk of contamination in the operating room. In addition, the nVisor ST60 HMD is very heavy (1.3 kg), which can be uncomfortable for a surgeon if required to wear the device for a long time. Therefore, we subsequently used HoloLens as the HMD. HoloLens is not only an HMD but is also a self-contained computer that has a built-in camera and sensors, and weighs only 579 g. In addition, HoloLens can be operated with simple hand gestures and voice commands instead of touch [10]. Therefore, the virtual model could be operated (including transparency adjustment and access to anatomical information) in real time while remaining sterile. However, it remained to be determined whether HoloLens-based vascular localization meets clinical requirements (error of localization less than 5 mm). The present study demonstrates that the HoloLens-based vascular localization system could precisely localize vessels with a minimum mean error of 1.35 mm (SD 0.43) and maximum mean error of 3.18 (SD 1.32), which were within the clinically acceptable range of 5 mm [11].

To maximally simulate the state of operation, the static errors were tested under different operating conditions, including different visual angles and different light intensities, and motion errors were also tested to simulate the typical movements of surgeons. None of the differences in the errors tested under different visual angles was statistically significant, and there was also no difference between the static error and motion error. Thus, the system has good robustness, and can remain stable and precise in different states. Since an opaque virtual model could block out real information, the virtual model should be displayed in a semitransparent state. Since light used in the operation could affect observation of the semitransparent virtual model, we also tested the static errors under different light intensities. No differences in the errors tested under different light intensities were found, whereas the difference in the errors tested with or without light was significant. Based on this finding, we recommend using HoloLens-based surgical navigation without light (ie, turn off the light when localizing the vasculature).

### Prospects

Modern medicine is developing toward personalized and precision treatment. Image-assisted surgical navigation systems could enable individual surgical planning and precise surgical procedures, which largely involve the use of virtual and augmented/mixed reality techniques. In virtual reality, the user is completely separated from the real world and is highly immersed in a virtual world, whereas in augmented/mixed reality, the virtual elements are overlaid onto the user’s reality and thus appear to be part of the real world. With a virtual reality–based surgical navigation system, the surgical instruments must be registered for projection into the virtual world, and the surgeon must switch the view from the virtual world to the real world to perform surgery. By contrast, with an AR-based surgical navigation system, the surgeon can see the virtual anatomical model and the real world simultaneously, so that the surgery can be performed directly without switching the view and surgical instrument registration. In recent decades, image-assisted surgical navigation systems have largely depended on virtual reality, but have gradually transferred to AR, which we believe will become the mainstream. Microsoft HoloLens could simplify and popularize the use of AR-based image-assisted surgical navigation systems, with potential benefits of low weight, less hardware, and gesture control.

Perforator flaps have been widely used in reconstructive surgery owing to their multiple advantages such as low morbidity at the donor site, good reconstruction and appearance of the recipient site, flexible design, and short postoperative recovery time [12]. Perforator dissection is a standard and important procedure for perforator flap transplantation, and also the most difficult process due to the uncertainty in predicting the anatomical location of the perforator and high variability in perforator size and course [13]. In this context, HoloLens-based perforator localization could play a pivotal role by offering individual 3D perforator information during the operation that is projected directly onto the patient with precise registration.

### Conclusion

The results of this study demonstrated that the HoloLens-based vascular localization system could precisely localize the perforator and might help a surgeon accomplish the operation. Although the precision of this system reached the clinical requirement, reduction of the error is still an important issue that deservers further study. The potential sources of error could be produced from the very beginning of the process from computed tomography/magnetic resonance imaging data acquisition through to the end of the surgical procedure, including imaging error, registration error, tracking error, and human error. Limitations of the hardware could also be a source of error, such as the perceptual limit of HMD [14]. Further study is ongoing and upcoming to confirm and seek solutions to eliminate or diminish these error sources. Clinical research will also be carried out to verify the feasibility of the system. Additionally, clinical application may encounter some problems such as the anatomical deformation between the CTA scan and operation, and the muscle relaxation caused by anesthesia. Thus, further research on precision is still needed, and a body fixation device may need to be integrated in the system to reduce the influence of anatomical deformation.

## Abbreviations

3D: three-dimensional
AR: augmented reality
CTA: computed tomographic angiography
HMD: head-mounted display
